# Borderline Values of Troponin-T and High Sensitivity C-Reactive Protein Did Not Predict 2-Year Mortality in TnT Positive Chest-Pain Patients, Whereas Brain Natriuretic Peptide Did

**DOI:** 10.3389/fcvm.2015.00016

**Published:** 2015-04-08

**Authors:** Dennis W. T. Nilsen, Øistein Rønneberg Mjelva, Ricardo A. Leon de la Fuente, Patrycja Naesgaard, Volker Pönitz, Trygve Brügger-Andersen, Heidi Grundt, Harry Staines, Stein Tore Nilsen

**Affiliations:** ^1^Department of Cardiology, Stavanger University Hospital, Stavanger, Norway; ^2^Department of Clinical Science, University of Bergen, Bergen, Norway; ^3^Department of Medicine, Stavanger University Hospital, Stavanger, Norway; ^4^Cardiology Research Institute, Catholic University of Salta, Salta, Argentina; ^5^Sigma Statistical Services, Balmullo, UK; ^6^Department of Research, Stavanger University Hospital, Stavanger, Norway; ^7^Department of Clinical Medicine, University of Bergen, Bergen, Norway

**Keywords:** chest-pain, acute coronary syndrome, total mortality, cardiac mortality, biomarkers, troponin-T, brain natriuretic peptide, high sensitivity C-reactive protein

## Abstract

**Background:**

Troponin-T (TnT), high-sensitive C-reactive protein (hsCRP), and Brain Natriuretic Peptide (BNP) have been shown to be independent prognostic indicators of total and cardiac death during short- and long-term follow-up.

**Methods:**

We investigated prospectively the prognostic value of admission samples of TnT, hsCRP, and BNP in 871 chest-pain patients from South-Western Norway and 982 patients from Northern Argentina, based on a similar protocol and database setup. Follow-up was 2 years for the pooled population. The prognostic value of the selected biomarkers was investigated in quartiles of 239 patients with TnT values greater than 0.01 and up to and including 0.1 ng/mL, with continuous TnT as a potential confounder.

**Results:**

After 24 months, 69 patients had died, of whom 38 died from cardiac causes. In the selected range of TnT, this biomarker was not significantly different between patients who died and survived (mean 0.0452 and 0.0457, *p* = 0.887). The BNP levels were significantly higher among patients dying than in long-term survivors [340 (142–656) versus 157 (58–367) pg/mL (median, 25 and 75% percentiles), *p* < 0.001]. In a multivariable Cox regression model for death within 2 years, the hazard ratio (HR) for BNP in the highest quartile (Q4) as compared to the lowest (Q1) was significantly related to total mortality [HR 2.84 (95% confidence interval (CI), 1.13–7.17)], *p* = 0.027, in addition to age (*p* ≤ 0.001) and hypercholesterolemia (*p* = 0.043). For cardiac death, the HR for BNP was 5.18 (95% CI, 1.06–25.3), *p* = 0.042. Several other variables (age, congestive heart failure, ST elevation myocardial infarction, and study country) were also significantly related to cardiac death. In a multivariable Cox regression model, hsCRP rendered no significant prognostic information for all-cause mortality (*p* = 0.089) or for cardiac mortality (*p* = 0.524).

**Conclusion:**

In patients with borderline TnT values (greater than 0.01 and up to and including 0.1 ng/mL), this biomarker as well as hsCRP did not render prognostic information, whereas BNP was found to be a strong prognostic indicator of 2-year total and cardiac mortality.

## Introduction

Several studies have employed troponins, BNP and hsCRP in risk stratification of patients presenting with an acute coronary syndrome (ACS). An early paper taking into account these three biomarkers in prognostication of later events had a follow-up of 10 months and was published by Sabatine et al. (1).

The troponins have become both a diagnostic and prognostic tool in ACS (2, 3). However, this biomarker is sensitive to myocardial damage, and so low serum values following an attack of chest-pain may not only indicate a primary coronary event, but may also be due to other conditions leading to its release (4). Thus, its prognostic implications may be masked by multifactorial causality when released in low concentrations. Accordingly, other biomarkers, such as hsCRP and BNP, may serve as better prognostic tools in low troponin release conditions.

BNP is a counter-regulatory peptide hormone predominantly synthesized in the ventricular myocardium. It is released into the circulation in response to ventricular dilatation and pressure overload (5, 6). BNP is an established biomarker of left ventricular dysfunction and heart failure (HF). It has also been shown to provide prognostic information beyond and above left ventricular ejection fraction (LVEF) in patients with ACS (7–13).

C-reactive protein (CRP) is an acute-phase reactant and a marker for underlying systemic inflammation, irrespective of its etiology, also reflecting atherosclerosis and plaque rupture (14–16), when applying high sensitivity CRP assays (17). However, the prognostic value of this marker in CVD is still controversial (18–20), although supported by our latest study (13).

The aims of this study were to explore the ability of BNP and hsCRP to predict risk of total and cardiac mortality within 24 months following the index event in a consecutively hospitalized chest-pain population with troponin release >0.01 and ≤0.1 ng/mL, pooling two populations with suspected ACS.

## Materials and Methods

This analysis is based on a two-center prospective follow-up study [Risk in the Acute Coronary Syndrome (RACS), ClinicalTrials.gov Identifier: NCT00521976 and Argentinean Risk Assessment Registry in ACS (ARRA-RACS), ClinicalTrials.gov Identifier: NCT01377402]. We recruited 871 consecutive chest-pain patients with suspected ACS in RACS and 982 patients in ARRA-RACS, consecutively admitted to the Emergency Department (ED) of Stavanger University Hospital, Stavanger, Norway, and to nine hospitals in Salta, Argentina, respectively. The same protocol and case report form (CRF) was used in both studies. Both studies were run between 2002 and 2011. The main exclusion criteria were age <18 years or unwillingness or incapacity to provide informed consent and prior inclusion in the present study. We used troponin-T (TnT) levels at baseline and at 6 h after admission for disease classification. BNP and hsCRP were determined in all patients as quality indicators in our registry.

Two-hundred and thirty nine patients with a troponin release >0.01 and ≤0.1 ng/mL were included in the present study. Patients with missing values for BNP, hsCRP, or a potential confounder were excluded from the multivariable Cox models. The primary and secondary outcome measure was defined as all-cause mortality and cardiac mortality, respectively. Follow-up time was 2 years from inclusion. Survival status, date and cause of death, and clinical data were obtained by telephone interview at 30 days, 6, 12, and 24 months during the 2-year follow-up period. In case of incapacity to provide information, the general practitioner or nursery home were contacted to obtain relevant data. In addition, hospital journals were searched for confirmation of reported data.

Clinical and laboratory parameters, including assessment of previous myocardial infarction (MI), angina pectoris, congestive heart failure (CHF), diabetes mellitus (defined as either whole blood fasting glucose concentrations above 6.1 mmol/L, 2 h post glucose load concentrations above 10.0 mmol/L, or medically treated diabetes mellitus), smoking status, hypercholesterolemia (defined as total cholesterol concentrations above 6.5 mmol/L or medical treated hypercholesterolemia), and arterial hypertension (defined as repeated blood pressure measurements above 140/90 mmHg or treated hypertension) were based on hospital records and personal interviews.

Electrocardiographic (ECG) findings at admission were classified according to the presence of ST-segment changes (i.e., ST-segment depression or elevation, T-wave inversion, or left bundle-branch block). The term ACS encompasses unstable angina (UAP), non-ST-segment elevation myocardial infarction (NSTEMI), and ST-segment elevation myocardial infarction (STEMI). In the present substudy, we included all subjects with a troponin release >0.01 and ≤0.1 ng/mL, irrespective of this classification. However, for the diagnosis of a MI, we applied a cut-off value of 0.05 ng/mL in the Norwegian population and 0.03 ng/mL in the Argentinean population.

Written informed consent was obtained from all patients. The study was approved by the Ethics Committee at the Board of Medical School of Salta, Argentina, and by the Regional Board of Research Ethics and the Norwegian Health authorities, and conducted in accordance with the Helsinki declaration of 1971, as revised in 1983.

## Blood Sampling Procedures and Laboratory Measurements

Peripheral blood samples for determination of TnT, s-creatinine, s-glucose, s-lipids, hsCRP, and BNP were drawn immediately following admission by direct venipuncture with a minimum of stasis of an antecubital vein. A repeated blood sample for the determination of TnT was drawn 6–7 h following the primary blood sample. Clotted whole blood and ethylene diamine tetraacetic acid (EDTA) blood samples were centrifuged for 15 min with 2000 × *g* at 20°C without delay. Serum for hsCRP and EDTA plasma for BNP were immediately frozen and stored at −80°C until the measurements were performed. For all other biochemical parameters, measurements were performed immediately following centrifugation.

Troponin-T was quantified in serum by a cardiac-specific third-generation (Norwegian population) and fourth-generation (Argentinean population) TnT ELISA assay from Roche Diagnostics, using a high-affinity cardiac-specific TnT isoform antibody (21). The lower limit of detection for the assays was 0.01 ng/mL. We selected borderline TnT values >0.01 and ≤0.1 ng/mL for prognostic evaluation.

BNP was analyzed in EDTA plasma using the microparticle enzyme immunoassay (MEIA) Abbott AxSYM^®^ (Abbott Laboratories, Abbott Park, IL, USA). The dynamic range was 0–4000 pg/mL and the within run coefficient of variation (CV) was 6.3% at 95 pg/mL and 4.7% at 1587 pg/mL.

High-sensitive C-reactive protein was measured with the use of an immunoturbidimetric assay [Tina-quant^®^ CRP (latex) high-sensitive assay, Roche Diagnostics, Germany] performed on a Roche automated clinical chemistry analyzer (MODULAR P). The detection limit was 0.03 mg/L and the measuring range 0.1–20.0 mg/L with an extended measuring range with automatic re-run 0.1–300 mg/L. The between-assay CV was 3.45% at 1.19 mg/L and 2.70% at 0.43 mg/L.

## Statistical Analysis

Patients were divided into quartiles according to their BNP and CRP levels. Approximately normally distributed variables were given as mean and standard deviation (SD) whilst quartiles were given for skewed continuous data. One way analysis of variance and the Kruskal–Wallis test were used to test for differences amongst groups of patients in the means of normally distributed and medians of skewed data, respectively. The Chi-square test for association was used to test for differences in proportions amongst groups of patients. The hazard ratios (HRs) comparing the lowest quartile to each of the other quartiles from Cox regression models are presented with 95% confidence intervals (CIs). Separate stepwise Cox multivariable proportional hazards regression models with total and cardiac death as the dependent variable were applied. The Cox regression model contained BNP and CRP quartiles. In addition, potential confounders added stepwise were: country, gender, age, smoking, hypertension, STEMI, diabetes mellitus, CHF (defined as Killip–Kimball class at admission; patients in class 2–4 were classified as CHF patients and patients in class 1 were classified as non-CHF), history of previous CHD [i.e., history of either angina pectoris, MI, CABG, or percutaneous coronary intervention (PCI)], hypercholesterolemia/use of statins, TnT, eGFR, and beta-blocker use prior to enrollment. Subjects without an event were censored at final follow-up. The Kaplan–Meier product limits were used for plotting the times to event with the equality of the survival curves assessed by the log-rank test. Hypothesis *p*-values less than 0.05 two-sided are defined as statistically significant.

## Results

A total of 239 patients presenting with chest-pain and suspected ACS were selected in the present substudy based on a TnT value >0.01 and ≤0.1 ng/mL. BNP or hsCRP samples were unavailable for eight and one patient, respectively. Death information was not available in one patient. In the Argentinean cohort, 59.6% had TnT values >0.03 ng/mL as compared to 60.3% in the Norwegian cohort. The corresponding proportion of patients with TnT values >0.05 ng/mL was 36.7 and 35.5%, respectively.

The median BNP concentration in plasma was 193 (66–442) pg/mL, 25 and 75 percentiles. The baseline characteristics of the patients, stratified according to BNP quartiles at admission are listed in Table 1. A TnT value >0.03 ng/mL was recorded in 151 subjects. TnT increased by at least 20% in 130 patients and by at least 50% in 93 individuals. A drop in TnT by at least 20% and 50% was noted in 51 and 10 patients, respectively. Peak TnT levels were similar in all quartiles of BNP. There were significant differences in the mean age and several other variables of the patients across the four BNP quartiles. Patients in the highest quartile were oldest on average. They were more often affected by previous heart disease and CHF and had a significantly lower eGFR as compared to patients in Q1. Their hsCRP was also increased and there was a higher proportion of Norwegians than Argentineans in the highest quartile of BNP.

**Table 1:** **Baseline characteristics according to BNP (pg/mL) quartiles**.

Variable	Total cohort ^a^	Q1 (*n* = 57)	Q2 (*n* = 59)	Q3 (*n* = 57)	Q4 (*n* = 58)	*p*-Value
Age, year^b^	72.5 ± 13.1	65.6 ± 12.4	71.7 ± 13.5	74.1 ± 13.1	78.7 ± 9.8	<0.001
Male gender, % (*n*)	61.9 (143)	64.9 (37)	67.8 (40)	54.4 (31)	60.3 (35)	0.473
Risk markers at baseline
Peak TnT (ng/mL)^c^	0.04 (0.02–0.06)	0.04 (0.03–0.07)	0.04 (0.02–0.07)	0.04 (0.02–0.06)	0.04 (0.03–0.06)	0.599
hsCRP (mg/L)^c^	6.3 (1.9–20.0)	3.4 (1.6–12.7)	3.5 (1.6–17.0)	7.8 (2.1–20.5)	9.2 (3.2–30.3)	0.016
BNP (pg/mL)^c^	193 (66–442)	35 (22–55)	117 (88–157)	295 (241–365)	707 (570–1196)	<0.001
Risk factors
Current smoking, % (*n*)	15.6 (36)	26.3 (15)	8.5 (5)	14.0 (8)	13.8 (8)	0.058
Hypertension, % (*n*)	57.1 (132)	52.6 (30)	55.9 (33)	59.7 (34)	60.3 (35)	0.826
IDDM, % (*n*)	1.3 (3)	1.8 (1)	0 (0)	3.5 (2)	0 (0)	0.283
NIDDM, % (*n*)	30.3 (70)	24.6 (14)	30.5 (18)	40.4 (23)	25.9 (15)	0.245
eGFR (mL/min/1.73m^2^)^b^	61.2 ± 28.8	76.1 ± 20.9	62.0 ± 31.6	57.3 ± 29.5	49.4 ± 25.7	<0.001
Hypercholesterolemia, % (*n*)	36.4 (84)	38.6 (22)	35.6 (21)	36.8 (21)	34.5 (20)	0.972
History of CHD
Angina pectoris, % (*n*)	42.0 (97)	36.8 (21)	39.0 (23)	45.6 (26)	46.6 (27)	0.650
MI, % (*n*)	28.1 (65)	10.5 (6)	25.4 (15)	42.1 (24)	34.5 (20)	0.001
CABG, % (*n*)	10.8 (25)	3.5 (2)	11.9 (7)	12.3 (7)	15.5 (9)	0.197
PCI, % (*n*)	10.8 (25)	10.5 (6)	11.9 (7)	10.5 (6)	10.3 (6)	0.993
History of heart failure
CHF, % (*n*)	34.2 (79)	19.3 (11)	27.1 (16)	38.6 (22)	51.7 (30)	0.002
Known CHD, % (*n*)	61.9 (143)	49.1 (28)	55.9 (33)	68.4 (39)	74.1 (43)	0.023
Treatment prior to admission
Beta blockers, % (*n*)	33.8 (78)	24.6 (14)	33.9 (20)	38.6 (22)	37.9 (22)	0.361
Index diagnosis
STEMI, % (*n*)	7.4 (17)	10.7 (6)	6.8 (4)	10.5 (6)	1.7 (1)	0.214
Country
Argentina, % (*n*)	47.2 (109)	57.9 (33)	61.0 (36)	42.1 (24)	27.6 (16)	0.001

*^a^Total cohort with available measurements: TnT; *n* = 230, hsCRP; *n* = 238, BNP; *n* = 231*.

*^b^Age, eGFR, total cholesterol, HDL cholesterol, and triglycerides are given as mean ± SD*.

*^c^Peak TnT, hsCRP, and BNP are given as median (quartiles Q1–Q3)*.

*TnT, troponin-T; hsCRP, high-sensitive C-reactive protein; BNP, B-type natriuretic peptide; IDDM, insulin-dependent diabetes mellitus; NIDDM, non-insulin-dependent diabetes mellitus; eGFR, estimated glomerular filtration rate; MI, myocardial infarction; CABG, coronary artery bypass graft; PCI, percutaneous coronary intervention; CHF, congestive heart failure; CHD, coronary heart disease; STEMI, ST-segment elevation myocardial infarction*.

The median hsCRP concentration in serum was 6.4 (1.9–20.0) mg/mL, 25 and 75 percentiles. The baseline characteristics of the patients, stratified according to hsCRP quartiles at admission are listed in Table 2. There were significant differences in several variables across the four hsCRP quartiles. Patients with hsCRP in the highest quartile were older and had a higher level of BNP and a lower level of eGFR, and the proportion of patients on β-blocker treatment was lower, as compared to Q1. A higher proportion of patients in Q1 as compared to the higher quartiles had undergone PCI.

**Table 2:** **Baseline characteristics according to hsCRP (mg/L) quartiles**.

Variable	Total cohort ^a^	Q1 (*n* = 58)	Q2 (*n* = 61)	Q3 (*n* = 59)	Q4 (*n* = 60)	*p*-Value
Age, year^b^	72.7 ± 13.0	70.6 ± 13.8	71.2 ± 11.1	75.3 ± 13.4	73.7 ± 13.2	0.171
Male gender, % (*n*)	62.2 (148)	63.8 (37)	62.3 (38)	64.4 (38)	58.3 (35)	0.904
Risk markers at baseline
Peak TnT (ng/mL)^c^	0.04 (0.02–0.06)	0.05 (0.03–0.07)	0.04 (0.03–0.06)	0.04 (0.02–0.06)	0.04 (0.02–0.06)	0.256
hsCRP (mg/L)^c^	6.35 (1.90–20.0)	1.2 (0.5–1.6)	3.1 (2.4–4.6)	11.2 (8.3–16.0)	46.2 (29.8–132.8)	<0.001
BNP (pg/mL)^c^	193 (65–443)	100 (51–283)	172 (64–372)	231 (72–473)	276 (104–567)	0.006
Risk factors
Current smoking, % (*n*)	15.5 (37)	12.1 (7)	16.4 (10)	10.2 (6)	23.3 (14)	0.200
Hypertension, % (*n*)	56.3 (134)	56.9 (33)	57.4 (35)	52.5 (31)	58.3 (35)	0.924
IDDM, % (*n*)	1.3 (3)	1.7 (1)	0 (0)	1.7 (1)	1.7 (1)	0.790
NIDDM, % (*n*)	29.8 (71)	24.1 (14)	31.2 (19)	33.9 (20)	30.0 (18)	0.702
eGFR (mL/min/1.73m^2^)^b^	61.3 ± 28.5	67.7 ± 27.9	64.4 ± 25.2	60.9 ± 27.6	52.3 ± 31.2	0.020
Hypercholesterolemia, % (*n*)	35.7 (85)	48.3 (28)	42.6 (26)	27.1 (16)	25.0 (15)	0.017
History of CHD
Angina pectoris, % (*n*)	42.4 (101)	39.7 (23)	49.2 (30)	49.2 (29)	31.7 (19)	0.154
MI, % (*n*)	28.2 (67)	25.9 (15)	31.1 (19)	32.2 (19)	23.3 (14)	0.662
CABG, % (*n*)	10.9 (26)	10.3 (6)	16.4 (10)	5.1 (3)	11.7 (7)	0.262
PCI, % (*n*) angina	10.1 (24)	15.5 (9)	18.0 (11)	0 (0)	6.7 (4)	0.004
History of heart failure
CHF, % (*n*)	35.3 (84)	25.9 (15)	27.9 (17)	44.1 (26)	43.3 (26)	0.060
Known CHD, % (*n*)	62.6 (149)	58.6 (34)	67.2 (41)	66.1 (39)	58.3 (35)	0.632
Treatment prior to admission
Beta blockers, % (*n*)	33.6 (80)	37.9 (22)	45.9 (28)	30.5 (18)	20.0 (12)	0.020
Index diagnosis
STEMI, % (*n*)	7.2 (17)	6.9 (4)	13.3 (8)	3.4 (2)	5.0 (3)	0.163
Country
Argentina, % (*n*)	45.4 (108)	50.0 (29)	47.5 (29)	37.3 (22)	46.7 (28)	0.529

*^a^Total cohort with available measurements: TnT; *n* = 230, hsCRP; *n* = 238, BNP; *n* = 231*.

*^b^Age, eGFR, total cholesterol, HDL cholesterol, and triglycerides are given as mean ± SD*.

*^c^Peak TnT, hsCRP, and BNP are given as median (quartiles Q1–Q3)*.

*TnT, troponin-T; HsCRP, high-sensitive C-reactive protein; BNP, B-type natriuretic peptide; IDDM, insulin-dependent diabetes mellitus; NIDDM, non-insulin-dependent diabetes mellitus; eGFR, estimated glomerular filtration rate; MI, myocardial infarction; CABG, coronary artery bypass graft; PCI, percutaneous coronary intervention; CHF, congestive heart failure; CHD, coronary heart disease; STEMI, ST-segment elevation myocardial infarction*.

## All-Cause Mortality

**Figure 1:**
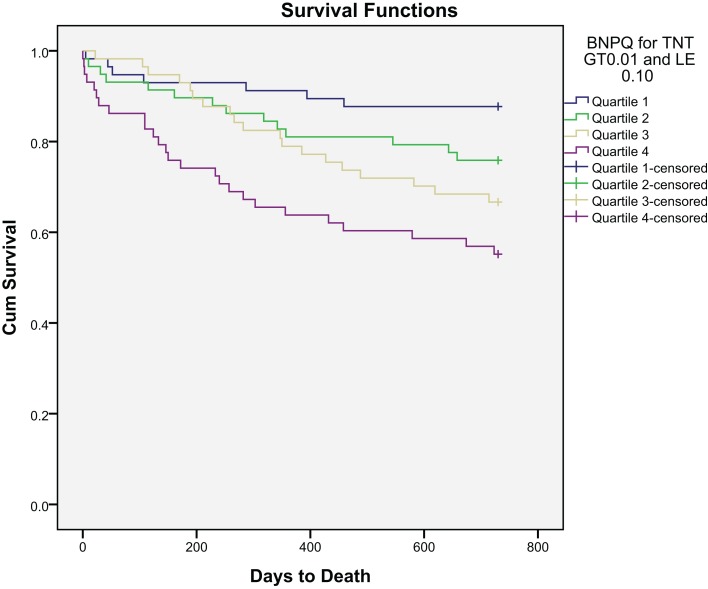
**Kaplan–Meier plot of time to all-cause mortality within 2 years by BNP quartiles**.

**Figure 2:**
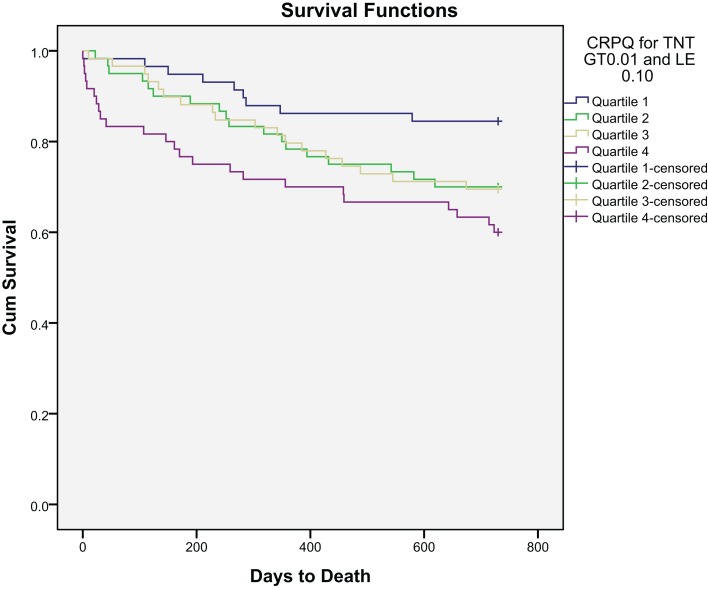
**Kaplan–Meier plot of time to all-cause mortality within 2 years by hsCRP quartiles**.

After a follow-up period of 24 months, 69 patients (29.0%) had died. Kaplan–Meier survival curves for the primary endpoint according to BNP and hsCRP quartiles at baseline are presented in Figures 1 and 2, respectively.

The BNP levels were significantly higher among patients dying than in survivors at 2 years [340 (142–656) versus 157 (58–367) pg/mL (median, 25 and 75 percentiles), *p* < 0.001]. hsCRP was also notably significantly higher among patients dying than in that of survivors at 2 years [8.9 (3.4–34.6) versus 4.8 (1.6–17.8) mg/mL (median, 25 and 75% percentiles), *p* = 0.004].

**Figure 3:**
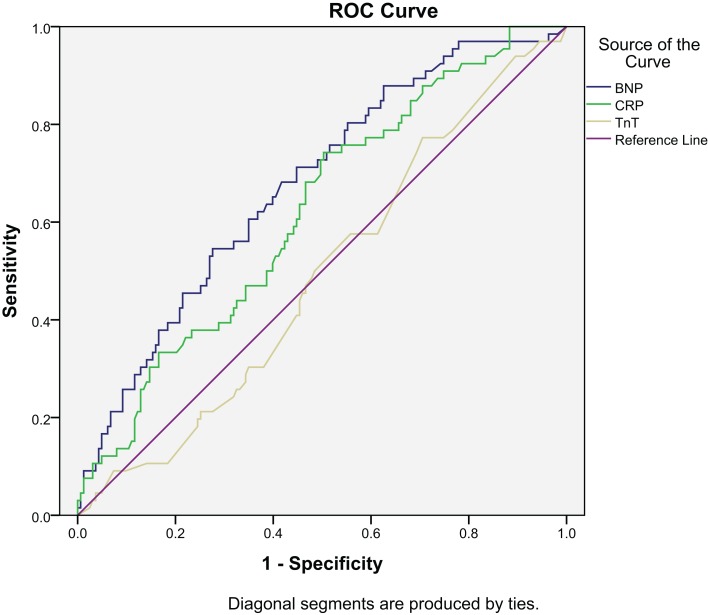
**ROC for all-cause mortality at 2 years: BNP, CRP, and TnT**.

Receiver operated characteristics (ROC) curve for BNP, hsCRP, and TnT are shown in Figure 3. The area under the ROC for BNP was 0.676 (*p* < 0.001) and for hsCRP 0.621 (*p* = 0.004), as compared to 0.489 (*p* = 0.803) for TnT.

The univariate HRs for total death were statistically significant for both BNP and hsCRP, as shown in Table 3.

**Table 3:** **Univariate and multivariate analysis**.

	All patients *n* = 239	
	Univariate^a^	Multivariate^a^
	HR (95% CI), *p*-value	HR (95% CI), *p*-value
BNP
Total death	4.60 (2.00–10.60), 0.001	2.84 (1.13–7.17), 0.027
Cardiac death	6.98 (2.05–23.85), 0.002	5.18 (1.06–25.26), 0.042
hsCRP
Death	3.10 (1.44–6.66), 0.004	2.00 (0.90–4.42), 0.089
Cardiac death	2.83 (0.995–8.02), 0.051	1.43 (0.48–4.25), 0.524

*^a^HR, hazard ratio; 95% CI, confidence interval. Highest quartile (Q4) versus lowest quartile (Q1). Cases with missing values in the multivariable analysis for total death, *n* = 12. Cases with missing values in the multivariable analysis for cardiac death, *n* = 20*.

In a multivariable Cox regression model for total death within 2 years, the HR for BNP in the highest quartile (Q4) as compared to the lowest (Q1) was 2.84 (95% CI, 1.13–7.17), *p* = 0.027 (Table 3), in addition to age [HR 1.05 (95% CI, 1.02–1.08)], *p* < 0.001 and hypercholesterolemia [HR 0.55 (95% CI, 0.31–0.98)], *p* = 0.043.

High-sensitive C-reactive protein rendered no significant prognostic information for all-cause mortality; HR for the highest versus the lowest quartile 2.0 (95% CI, 0.90–4.43), *p* = 0.089 (Table 3).

## Cardiac Death

**Figure 4:**
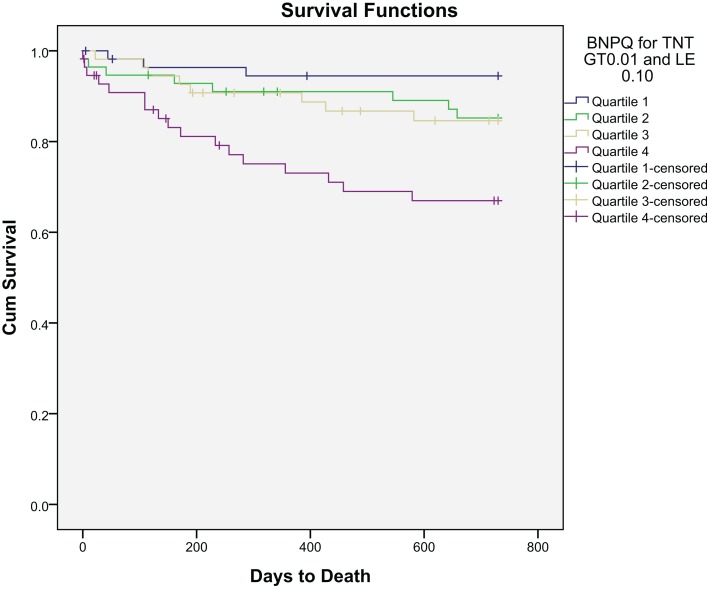
**Kaplan–Meier plot of time to cardiac death within 2 years by BNP quartiles**.

**Figure 5:**
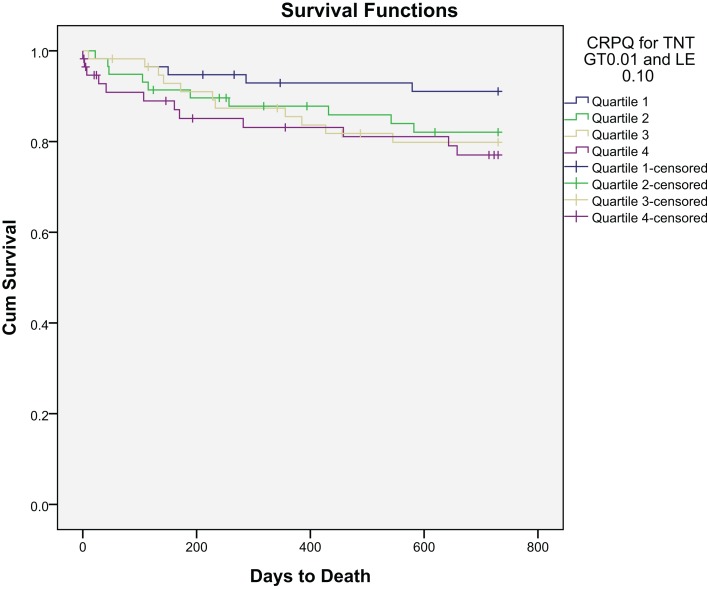
**Kaplan–Meier plot of time to cardiac death within 2 years by hsCRP quartiles**.

After a follow-up period of 24 months, 38 deaths (16.5%) were defined as cardiac. Kaplan–Meier survival curves according to BNP and hsCRP quartiles at baseline are presented in Figures 4 and 5, respectively.

**Figure 6:**
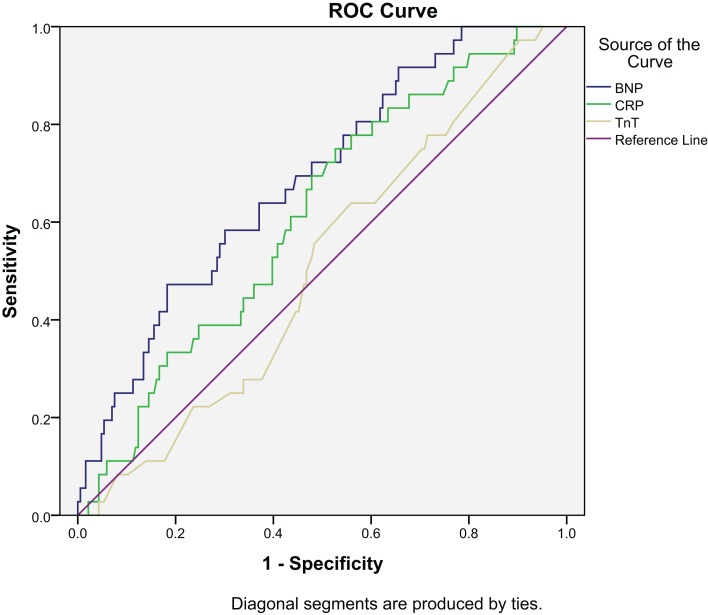
**ROC for cardiac death at 2 years: BNP, CRP, and TnT**.

The ROC curves for BNP, hsCRP, and TnT are shown in Figure 6. Area under the ROC for BNP was 0.687 (*p* < 0.001) and for hsCRP, it was 0.614 (*p* = 0.031), as compared to 0.506 (*p* = 0.903) for TnT.

The univariate HRs for cardiac death was statistically significant for BNP and borderline significant for hsCRP, as shown in Table 3.

In a multivariable Cox regression model for cardiac death within 2 years, the HR for BNP was higher than for total mortality [HR 5.18 (95% CI, 1.06–25.3)], *p* = 0.042 (Table 3). Age [HR 1.12 (95% CI, 1.06–1.17), *p* < 0.001], CHF [HR 3.59 (95% CI, 1.56–8.27), *p* = 0.003], STEMI [HR 4.38 (95% CI, 1.09–17.60), *p* = 0.037], and country [HR 0.28 (95% CI, 0.11–0.69), *p* = 0.006] also predicted outcome.

In a multivariable Cox regression model, hsCRP rendered no significant prognostic information for cardiac mortality [HR 1.43 (95% CI, 0.48–4.25)], *p* = 0.53 (Table 3).

## Discussion

Continuous TnT values within the diagnostic borderline zone of TnT yielded no additional prognostic information in the present study, both in the univariate analysis, as reflected by the ROC curve, and by the following multivariable analysis, whereas BNP was found to be a substantial predictor of total and cardiac mortality.

Samples for TnT measurement were obtained at admission and 6 h following hospitalization, providing a definite diagnosis of myocardial injury (22–24). The highest TnT value within the defined borderline zone was used for assessment of its prognostic utility for future total and cardiac mortality. TnT increased by at least 20% in more than a half of the population and dropped by at least 20% in approximately 1/5 of the subjects. Thus, TnT dynamics of this magnitude was present in approximately 75% of the pooled population. A stable elevation of TnT in the remaining patients may reflect more chronic conditions.

As early as 1992, the prognostic value of TnT as compared to creatine kinase isoenzyme MB (CKMB) activity, was convincingly demonstrated in UAP (25). In that study, TnT was detected in the range of 0.20–3.64, median 0.50 ng/mL, which is above the studied range in the present study. They employed a test kit with a sensitivity of 0.20 ng/mL, whereas we used a TnT kit with a sensitivity of 0.01 ng/mL. Several studies have shown that the level of TnT contributes to risk stratification (26, 27). However, borderline values of TnT may reflect a variety of conditions, and sensitivity and specificity is a challenge.

In a recent paper by Haaf and coworkers (23), high-sensitive troponin-T (hsTnT) and fourth-generation TnT predicted 2-year mortality in a prospective, international multicenter study including 1117 unselected patients with chest-pain, of whom 82 (7.3%) died during the observation period. In their study, blood was collected at presentation to the ED and serially thereafter at 1, 2, 3, and 6 h, whereas in our two pooled studies, conventional TnT was measured at presentation to the ED and 6 h thereafter. In comparison, our pooled cohort consisted of 1853 patients and during our 2-year follow-up, 257 (13.9%) patients had died. In the paper by Haaf et al. (23), the risk of patients with negative conventional TnT values, but elevated hsTnT at presentation, was found to be significantly increased. The prognostic pattern of hsTnT seems to differ from that of conventional TnT, as its association with mortality seems to be linear (23).

In the present study, we have focused on the prognostic utility of conventional TnT (third- and fourth-generation) in chest-pain patients with borderline (0–6 h) values of TnT (>0.01 and ≤0.1 ng/mL). Within this restricted interval, continuous TnT values offered no additional prognostic information. Our subset of patients with borderline TnT values consisted of 239 subjects of whom 69 (29%) had died at 2-year follow-up, exceeding the proportion of patients dying in the corresponding conventional TnT zone in the study by Haaf et al. (23).

Elevated troponins indicate cardiac injury, but do not define the cause of the injury (4). Thus, borderline TnT values ≤0.1 ng/mL are not restricted to coronary events, but may also represent other conditions such as stroke, pulmonary embolism (PE), sepsis, acute perimyocarditis, Tako-tsubo, acute HF, and tachycardia.

Two-year mortality in our subset of patients with borderline TnT was not predicted by the level of this myocardial injury biomarker, which clearly confirms the importance of adding other biomarkers for risk assessment in this zone of TnT. Thus, by adding BNP and hsCRP, the former was found to be independently associated with both total and cardiac mortality in our quartile multivariable model. Furthermore, we found that the prognostic utility of hsCRP reached statistical significance in the univariate analysis, but this biomarker did not behave as an independent predictor in the multivariable analysis.

Our results are in line with the recommendations in the ESC guidelines (25). As stated in these guidelines, cardiac troponins are the key biomarkers for initial risk stratification, whereas other biomarkers, such as BNP (28, 29) and hsCRP (30, 31), may provide incremental prognostic information.

BNP is not only released in HF (6–8), but also by myocardial ischemia (32–34) and by lung embolism (35). Retrospective data in patients with NSTE-ACS (29) show that elevated BNP or NT-proBNP levels are associated with a significantly increased mortality rate as compared to lower levels, independent of troponin and hsCRP measurements.

Cut-off levels of BNP vary according to presenting symptoms and age. A cut-off value of 100 pg/mL has a strong negative predictive value, ruling out a diagnosis of HF (36).

In the present prospective study, BNP values in the upper quartile(s) were clearly elevated and the rate of CHF was almost three times as high in the upper as compared to the lower quartile. In fact, there was a graded increase in CHF across the quartiles of BNP from Q1 to Q4. These findings are expected, as BNP is a confirmed marker of heart insufficiency. However, as BNP was found to be an independent marker of survival, other factors, as mentioned above, may contribute. Thus, natriuretic peptide measurements on admission or during hospitalization are useful for risk stratification irrespective of the cause (24, 37).

Limitations: our results are limited to two chest-pain populations, from Norway and from Argentina, respectively. Both BNP and hsCRP are based on one single blood sample obtained upon admission, whereas TnT was measured twice, at admission and 6 h later. We did not include recordings of LVEF in this study. Patients with CHF included patients with known HF, categorized by NYHA class II–IV (12), or those with Killip class 2–4 on admission, as previously described (13). Patients with CHF accounted for 35.3% of the population. Both BNP and hsCRP values in the present study mirror high age as well as a higher burden of disease, including CHF and reduced renal function.

Inclusions and follow-up were based on the same CRF and the 2-year follow-up program was completed within the same decade. We have not presented further diagnostic and angiographic information, as the aim of this study was to investigate the prognostic contribution of the studied biomarkers, irrespective of etiology.

As reperfusion therapy in STEMIs may influence outcome, this should be mentioned. Only 42% of the total Argentinean STEMI cohort received this treatment (13) as compared to the general use of reperfusion therapy in Norway. As only 7.1% of the pooled patients in the present study presented with a STEMI, the proportion of patients not receiving reperfusion therapy was low and of less importance to the interpretation of results.

In conclusion, TnT may be a useful tool for stratifying patients at risk, but borderline admission levels of this biomarker were not found to yield additional prognostic information during a 2-year follow-up period. However, within the selected TnT range, the upper as compared to the lower quartile of BNP predicted total and cardiac mortality after adjusting for covariables, whereas hsCRP was not found to be an independent predictor in our multivariable model. BNP should be assessed routinely in patients with borderline TnT values and an increase in BNP should lead to further clinical investigation.

## Conflict of Interest Statement

The authors declare that the research was conducted in the absence of any commercial or financial relationships that could be construed as a potential conflict of interest.
